# Cost of antenatal care for the health sector and for households in Rwanda

**DOI:** 10.1186/s12913-018-3013-1

**Published:** 2018-04-10

**Authors:** Regis Hitimana, Lars Lindholm, Gunilla Krantz, Manassé Nzayirambaho, Anni-Maria Pulkki-Brännström

**Affiliations:** 1 Epidemiology and Global Health, Department of Public Health and Clinical Medicine, Umeå University, 901 87 Umeå, SE Sweden; 2 School of Public Health, College of Medicine and Health Sciences, University of Rwanda, Kigali, Rwanda; 3 Section of Epidemiology and Social Medicine (EPSO), Department of Public Health and Community Medicine, The Sahlgrenska Academy, University of Gothenburg, Gothenburg, Sweden

**Keywords:** Antenatal care, Cost of care, Rwanda

## Abstract

**Background:**

Rwanda has made tremendous progress in reduction of maternal mortality in the last twenty years. Antenatal care is believed to have played a role in that progress. In late 2016, the World Health Organization published new antenatal care guidelines recommending an increase from four visits during pregnancy to eight contacts with skilled personnel, among other changes. There is ongoing debate regarding the cost implications and potential outcomes countries can expect, if they make that shift. For Rwanda, a necessary starting point is to understand the cost of current antenatal care practice, which, according to our knowledge, has not been documented so far.

**Methods:**

Cost information was collected from Kigali City and Northern province of Rwanda through two cross-sectional surveys: a household-based survey among women who had delivered a year before the interview (*N* = 922) and a health facility survey in three public, two faith-based, and one private health facility. A micro costing approach was used to collect health facility data. Household costs included time and transport. Results are reported in 2015 USD.

**Results:**

The societal cost (household + health facility) of antenatal care for the four visits according to current Rwandan guidelines was estimated at $160 in the private health facility and $44 in public and faith-based health facilities. The first visit had the highest cost ($75 in private and $21 in public and faith-based health facilities) compared to the three other visits. Drugs and consumables were the main input category accounting for 54% of the total cost in the private health facility and for 73% in the public and faith-based health facilities.

**Conclusions:**

The unit cost of providing antenatal care services is considerably lower in public than in private health facilities. The household cost represents a small proportion of the total, ranging between 3% and 7%; however, it is meaningful for low-income families. There is a need to do profound equity analysis regarding the accessibility and use of antenatal care services, and to consider ways to reduce households’ time cost as a possible barrier to the use of antenatal care.

**Electronic supplementary material:**

The online version of this article (10.1186/s12913-018-3013-1) contains supplementary material, which is available to authorized users.

## Background

Maternal health has improved in the last 25 years. Globally, maternal deaths have fallen by almost 44% since being included in the Millennium Development Goals in 1990. However, low-income countries still bear a big share of the global burden of maternal health problems. Sub-Saharan Africa alone accounted for 66.3% of world maternal deaths in 2015 [1], and 12 countries of the region still had more than 500 deaths per 100,000 livebirths compared to less than 5 deaths per 100,000 livebirths in the ten countries with the lowest maternal mortality rates [1].

Antenatal care is considered one of the safe motherhood interventions [2]. It consist of routine check-ups for mostly healthy pregnant women in order to identify signs and risks of disease and provide timely response [3]. In the early 1900’s, antenatal care was introduced in high-income countries, and later spread to other countries without strong evidence on its effectiveness with regard to content, number and timing of visits [4]. However, during the last few decades there has been a growing interest in documenting the effectiveness of antenatal care especially in low-income countries [5].

Rwanda has adopted the 2001 World Health Organization (WHO) model of four focused antenatal care visits for pregnant women without past and current complications. The country has made tremendous progress in maternal and child health, and is counted among the few countries that have achieved the fifth maternal health-related Millennium Development Goal [1], although the maternal mortality ratio is still high at 210 deaths per 100,000 livebirths [6]. Improved access and coverage of antenatal care have almost certainly played a role in improvements to maternal and newborn health in Rwanda. Nearly all (99%) women attend at least one antenatal care visit. However, less than half (44%) complete the four standard visits and only 56% of women go for their first visit before the 4th month of pregnancy [6].

In November 2016, WHO published new antenatal care guidelines that recommend eight visits or contacts with skilled personnel during pregnancy [7]. The debate as to whether it is worthwhile for low-income countries to mobilize the resources necessary to double the number of visits/contacts was immediately initiated in the Lancet issue of November 26th, 2016 [8]. To answer the question above, there is a need to understand the cost implications and potential outcomes countries can expect from adopting the new guidelines, as well as their individual abilities to mobilize the required resources. For Rwanda, a necessary starting point is to understand the cost of current antenatal care practice, which, according to our knowledge, has not been documented so far.

Existing literature on the cost of maternal health interventions in general and antenatal care in particular is characterized by wide variation in results. For example, a costing study conducted in three African countries found that the unit societal cost (household + health facility) of one antenatal care visit ranged between $2.2 and $6.4 in Uganda, between $3.2 and $5.8 in Malawi, and between $3 and $5.5 in Ghana. The main reason for variation in unit cost in these countries was the type of provider (hospital versus health center) and ownership (mission versus public) [9]. Another study estimated the mean societal cost of antenatal care (comprising provider costs, user time, and out-of-pocket expenditures) during the whole pregnancy period (four visits) to equal $1076 in Cuba and $194 in Thailand [10]. Such variation in the cost of antenatal care within and between countries suggests that the efficiency of service provision can be increased. For Rwanda, where only 36% of the total health budget is covered by domestic resources [11], it is extremely important to maximize efficiency, especially as foreign development assistance is unpredictable and being reduced over time. The aim of this study is to estimate the current cost of antenatal care services from the health care provider and household perspectives in Rwanda. The findings are intended to contribute to the wider literature on the cost of antenatal care in low-income countries, and specifically to the policy debate in Rwanda in terms of the efficiency of resource use and the key cost drivers in antenatal care services.

This study is part of the Maternal Health Research Program (MaTHeR) undertaken by the University of Rwanda in collaboration with Gothenburg University and Umeå University with funding from the Swedish International Development Agency.

## Methods

### Study setting and context

In the Rwandan public healthcare system, the lowest level of health care is the health post, followed by health centers, district hospitals, provincial hospitals and finally national referral hospitals. The national guidelines for antenatal care in Rwanda are based on the 2001 WHO model of four focused antenatal care visits for normal pregnancies. Standard antenatal care is provided at the health center. District hospitals are concerned with gynecological consultations for women with diagnosed complications and referrals from health centers. Most referral cases require an ultrasound investigation, which is not routinely provided for normal pregnancies. Health centers cover 20,000 to 25,000 persons, are run by nurses, and employ on average between eight and twenty nurses and midwives. The private sector is dominated by small dispensaries (also run by nurses) with a limited package of activities. In addition there are private clinics and polyclinics, mainly in the cities.

Rwandan Demographic and Health surveys have consistently shown that there are no major geographical differences in key reproductive health indicators apart from rural-urban patterns [6, 12, 13]. The present study was conducted in two provinces: Northern Province (rural) and Kigali city (urban). In the two provinces there are 151 public health facilities. Of these, three are national referral hospitals, eleven are district hospitals, two are specialized hospitals, and the remaining are health centers.

### Aim and overall study design

The aim of this study is to determine the current societal cost of antenatal care services in Rwanda. The societal perspective in this study encompasses the healthcare system and households. Costs incurred by providers comprised human resources, drugs and consumables, equipment, and infrastructure. Household costs considered were the cost of travel and time spent visiting the antenatal service. The cost information was collected from two sub-surveys: a cross-sectional health facility survey in six health facilities, and a household-based survey consisting of retrospective analysis of cohort information collected among women who had delivered a year before the interview. The resources used in each antenatal care visit (visit one, two, three and visit four) according to the Rwandan national guidelines were separately estimated. The cost calculation was done in Rwandan francs and converted to United States dollars using the 2015 annual average exchange rate from the National Bank of Rwanda (1 USD = 721 Rwf) [14].

### Health facility costing

The healthcare cost of ANC was collected from two facilities in Kigali city and four facilities in Northern province (Table 1). One of the facilities in Kigali city was a private tertiary hospital. All other facilities were either public or faith-based primary healthcare centers. In selecting health facilities, ownership was assumed to be the main potential cause of variation in the cost of antenatal care services. Table 1 shows that the estimated total number of antenatal care visits received in the six health facilities for the year 2015 varies between 2833 (Rutare Health center) and 20,022 (Hopital Croix du Sud).

**Table 1:** Description of selected health facilities

Name of facility	Province	Type of facility	Ownership	Number of women attending first ANC visit (2015)	Estimated annual number of ANC visits
Hopital Croix du Sud	Kigali	Polyclinic, tertiary care	Private	5985	20,022
Muhoza Health Centre	North	Primary health care	Public	3454	11,555
Masaka Health Centre	Kigali	Primary health care	Faith-based	1550	5185
Nemba Health Centre	North	Primary health care	Faith-based	1184	3961
Kinigi Health Centre	North	Primary health care	Public	1004	3359
Rutare Health Centre	North	Primary health care	Public	847	2833

Health facility data were collected in the period December 2014 to January 2015 using a structured data collection template (Additional file 1). A micro costing approach, which involves identification of resources used to provide the service, measuring, and valuing them [15], was used to collect health facility data. Interviews with staff responsible for ANC in each of the six health facilities was used to establish a list of detailed tasks performed during each of the four ANC visits. This list included the staff involved, their time use, consumables, equipment, and space required to perform each activity. Staff responsible for the laboratory was interviewed to identify and quantify laboratory-specific ANC resources. Finally, in each health facility, an accountant was approached to estimate the unit price of all inputs as well as overhead costs.

#### Cost of human resources

In each facility, interviews with the staff responsible for ANC were conducted in order to estimate the cost of human resources. The following information was collected: the number of staff directly involved in ANC tasks their position, qualifications, and annual gross salary; the days of the week that ANC service is provided in the facility; and the average number of hours that ANC activities take each day. The above elements enabled calculation of the proportion of person-hours and salary attributable to ANC activities, and furthermore the unit cost of each typical visit (ANC visits one, two, three and four).

#### Cost of consumables and equipment

Consumables and equipment used in each typical ANC visit (visit one, two, three and four) were identified and quantified based on the list of activities. They included reagents used in laboratory tests, drugs, vaccines, and gynecological beds.

The total annual cost of consumables for each visit in a given health facility was obtained by multiplying the unit cost (cost of one visit) by annual ANC utilization numbers for that facility (number of women/couples in visit one, two, three and four). Unit costs for subsidized consumables in public health facilities (mainly HIV test and malaria prevention) were collected from the central medical store with the value at service delivery level.

The total value of equipment was annuitized to give an estimate of the 2015 annual value. The time that equipment was used for ANC activities was applied to that annual value to provide an estimated cost of ANC equipment.

#### Cost of buildings

The standard building cost for a public health facility was used to estimate cost of buildings because actual information about the specific health facilities was lacking. The total cost was annuitized using 20 life years as the expected age of a new building, which is commonly used in depreciation calculations in accounting systems in Rwanda. The space dedicated to ANC was directly measured during data collection and applied to the total space for a public health facility to calculate the percentage of total building cost allocated to ANC in each health facility.

#### Overhead cost estimation and allocation

The overhead cost included non-clinical staff, shared equipment (e.g. cold chain equipment, power generators), and the cost of general supplies and utilities. Overhead costs were allocated using a direct allocation method with the share of staff-hours as the allocation basis, i.e. the proportion of ANC paid staff-hours against the total paid staff-hours at the facility (Additional file 1).

### Household costing

Household cost information was collected as part of a bigger household survey focusing on maternal and child health issues. The survey questionnaire was administered to 922 randomly selected women in the Northern province and Kigali city between July 2014 and January 2015. Households were selected in a multistage random sampling process. In a first step, 48 primary sampling units (villages, the lowest administrative entity in Rwanda) were selected from a list of all villages in the two provinces. The 48 villages correspond to 1 % of the total number of villages in the selected area. In a second step, the number of households to be visited in each village was selected proportionally to the total number of households in each village. The registry of households with a woman who gave birth in the past 13 months (kept by community health workers) was used to randomly select the households to be visited.

#### Description of the study population

The majority of the participating women lived in rural areas (77%). The main occupation for women was farming (69%). A further 7% were housewives, 4% had a paid job, and 9% were unemployed. The majority (88%) of women were 35 years old or less. Only 25% of the women were educated above primary school level. About half (53%) of the women were married; 32% were cohabitant; 2% separated, divorced or widowed; and 13% were single/not married (Additional file 2). Nearly all (95%) of the women/couples visited public/faith-based health centers for antenatal care.

#### Household cost of ANC

Additional file 3 provides an extract from the household questionnaire with the questions used in the present study. Information was collected on: the time it usually takes to travel to the ANC clinic, get the service and come back home; the means of transport; the typical cost of transport; the number of visits; whether the woman was accompanied or not; household monthly income; and the occupation of both the woman and the partner. The total cost of transport incurred by households was calculated by multiplying the median cost of transport by the proportion of women/couples who reported paying for transport when attending antenatal care. If the woman was accompanied, the cost of transport was doubled. To value the time spent to attend ANC for the women and those accompanying them, it was assumed that they would be occupied by their usual activities if not attending ANC. Occupation was categorized into paid employment, self-paid, and no employment. Women’s time use was valued by applying the national median daily income for the two categories (paid job and self-paid) from the 2016 National Labor Force Survey [16]. In cases where the woman was accompanied, the cost of time was doubled.

ANC service is subsidized in public health facilities, but paid for by users in private health facilities. In order to avoid double counting, fee payment by households was disregarded because it was intended to pay for inputs already counted, and in some cases, the profit of private health facilities. Our data suggest that 14% have paid for ANC service in health facilities.

### National cost of antenatal care

The cost of antenatal care for the whole of Rwanda was calculated by multiplying the average cost of all four visits in public and faith-based health facilities by the national ANC utilization figures from the national health information system [17]. This information system has recorded 373,678 women/couples who attended the first visit. Based on this, the number of subsequent visits was estimated assuming the same attendance rates as in our household survey.

The cost of ANC services delivered at private clinics was not included in the calculation of the national cost for two reasons: first, there was a large difference in the unit cost between public and private facilities; and second, because nearly all (95%) women/couples visited public/faith-based health centers for antenatal care.

## Results

### Cost of antenatal care for the health sector

The cost of the package of four antenatal visits per woman in the six health facilities is presented in Table 2. Hospital Croix du Sud (the private clinic) presented the highest cost at $137 per four ANC visits, followed by Nemba health center ($55) and Masaka health center ($47). Muhoza health center had the lowest cost at $33.

**Table 2:** Health facility cost of antenatal care

	Cost per visit (USD)				
Name of facility	1st visit	2nd visit	3rd visit	4th visit	Sum (4 visits)
*Private health facility*					
Croix du Sud hospital	73.5	27.0	24.2	12.3	137.0
*Public health facilities*					
Muhoza Health Centre	16.8	4.1	4.1	7.6	32.6
Masaka Health Centre	23.4	5.4	5.4	12.8	47.1
Nemba Health Centre	24.2	8.2	8.2	14.9	55.4
Kinigi Health Centre	20.6	6.0	6.0	10.3	42.8
Rutare Health Centre	18.5	4.2	4.2	7.9	34.9
*Average (mean) cost across public and faith based facilities*	*20.7*	*5.6*	*5.6*	*10.7*	*42.6*

The first visit had the highest unit cost in all facilities, ranging between $73 (Hospital Croix du Sud) and $ 17 (Muhoza Health Center). The 4th visit came second in cost for the public health facilities, ranging between $15 (Nemba Heath Center) and $8 (Mohoza and Rutare Health Centers). In Hospital Croix du Sud, the 2nd visit had the second highest unit cost at $27. In public facilities, the 2nd and 3^rd^visits were similar in cost ($8 - $4) and considerably lower than the other two visits.

### Cost of antenatal care for households

The estimated cost of antenatal care for households is presented in Table 3. The total time cost for women/couples to attend four visits was $3.34. The total cost of transport was $0.63. The first ANC visit had the highest cost ($1.5) in terms of both transport and time, while the three other visits were equal and considerably lower in cost ($0.82–0.84).

**Table 3:** Household cost of antenatal care

	Cost per visit (USD)				
	1st visit	2nd visit	3rd visit	4th visit	Sum (4 visits)
Transport	0.24	0.13	0.13	0.13	0.63
Time cost	1.25	0.71	0.69	0.69	3.34
Total	1.49	0.84	0.82	0.83	

### Societal cost of antenatal care in Rwanda

Adding up health facility and household costs, the societal cost of antenatal care was estimated to equal $160 per package of four visits in the private health facility and $44 in public health facilities (Table 4). The first visit cost $21 in public health facilities, considerably more than the following three visits ($6 - $11). In the private clinic, the cost of the first visit was $75 while the other three visits ranged between $28 and $29.

**Table 4:** Societal cost of antenatal care

	Annual total cost (USD)					
	1st visit	2nd visit	3rd visit	4th visit	Sum (4 isits)	%
Private health facility						
Personnel	108,393	79,254	70,897	24,757	283,302	34%
Drugs and consumables	301,336	60,352	54,531	42,150	458,369	54%
Equipment	2396	1752	1567	547	6262	1%
Cost of building	969	709	634	221	2533	0%
Overheads	26,614	19,459	17,407	6079	69,559	8%
Transport (households)	1408	1373	1228	643	4653	1%
Time cost (households)	7485	4138	3595	1899	17,117	2%
Total cost (1 facility)	448,601	167,037	149,859	76,296	841,794	100%
Annual number of visits^a^	5985	5835	5220	2734	19,773	
Cost per visit	75	29	29	28	160	
Public and faith based health facilities						
Personnel	18,868	7358	6582	5516	38,324	13%
Drugs and consumables	131,700	30,188	27,005	28,871	217,764	73%
Equipment	1037	404	362	303	2107	1%
Cost of building	969	709	634	221	2533	1%
Overheads	5833	2275	2035	1705	11,848	4%
Transport (households)	1892	1046	908	480	4326	1%
Time cost (households)	10,054	5559	4828	2551	22,992	8%
Total cost (5 facilities)	170,353	47,538	42,354	39,648	299,893	100%
Mean total cost per facility	34,071	9508	8471	7930	59,979	
Annual number of visits^a^	8039	7837	7011	3672	26,559	
Cost per visit	21	6	6	11	44	
National ANC utilization	373,678	364,299	325,885	170,696	1,234,557	
Estimated total national cost	7,918,560	2,209,701	1,968,732	1,842,977	13,939,970	

^a^The number of 1st visits refers to the year 2015

Table 4 also shows the breakdown of costs by type of input. Drugs and consumables were the main input category, accounting for 73% and 54% of the total cost in public and private health facilities respectively. Human resources came in the second position, accounting for 13% and 34% of the total cost in public and private health facilities respectively.

The national annual cost of ANC in the whole country in 2015 was estimated to be $13,939,970.

## Discussion

The average societal cost per ANC visit for the year 2015 in Rwanda varied between $11 and $40 in this study, for the public and private health facilities respectively. The comparison of this ANC cost with other published studies is complicated by the fact that few studies have included a societal perspective. Furthermore, there is variation in the content of the antenatal care package over time and across countries. Among studies that estimated ANC societal cost, cost estimates from other sub-Saharan African countries have been lower than the public facility estimates ($11–$21 per visit) obtained in the current study: in Uganda, the cost of one ANC visit was varying between $2.2 and $6.40, in Malawi between $3.2 and $5.8, in Ghana between $3 and $5.5 [9]. In contrast the estimates from other countries have been considerably higher: $215 for Cuba, $39 for Thailand [8] and $ 35.8 in Argentina [18]. Studies that have considered a provider perspective presented results close to those of this study and to each other: the mean cost of antenatal care was $16 in Tanzania [19], $18 in Ghana [20], and $18.78 in Pakistan [21].

Drugs and consumables was the main contributor to the total ANC cost. This was consistent with results of Livin et al. [9], who reported drugs and consumables as the main contributors to total ANC cost. However, other studies have reported human resources as the most costly input to antenatal care [18, 19, 21]. More generally, personnel is often reported as the input with the highest cost in primary health care [20, 22–24]. The type of ANC model implemented and the technological level used in laboratory tests in Rwanda can explain this pattern. The Rwandan ANC model (which follows the 2001 WHO model [10]) is dominated by clinical consultations (staff time), screenings and immunization activities, and a number of laboratory tests. Most laboratory tests are conducted during the first visit and a majority of those tests (e.g. pregnancy test, HIV, syphilis) are rapid tests, which require reagents (classified in this study in drugs and consumables) and less laboratory equipment. Alike many other low-income countries, where malaria and HIV are major public health problems [25], Rwanda has followed recommendations to integrate the screening and prevention of those diseases into antenatal care and mostly during the first visit. The mosquito nets distribution and laboratory tests during the first visit that are not repeated in follow-up visits also contributed to the higher cost of the first visit for health facilities. For households, the first visit was most expensive because partners typically accompany women for this visit, e.g. for the purpose of HIV testing.

The results of this study suggest that the unit cost of providing ANC services is much lower in public health facilities compared to a private health facility. The average cost of the package of four antenatal care visits during pregnancy was $44 across the five public facilities and $160 in the private facility. A similar finding was documented in India, where the cost of antenatal care provision in private health facilities was more than double the cost of public sector provision [26]. In our study, the higher cost of ANC in the private clinic can be partly explained by the highly qualified and better-remunerated staff compared to public health facilities surveyed in this study. While the public health facilities generally appeared to follow the national guidelines, antenatal care practice in the private clinic differed in three significant ways. First, the ANC consultation was performed by a gynecologist, while in the public facilities consultation was done by nurses and midwives. Second, the private clinic performed extra laboratory tests, such as screening for rubella, hepatitis B, and hepatitis C. Third, all women were offered up to three ultrasound scans. In contrast, the public health centers did not routinely use ultrasound. In this study, we have excluded those activities performed by the private facility that go beyond the national ANC guidelines for normal pregnancy, in order to facilitate comparison with the public facilities. Those activities include all ultrasound examinations (at $3.03 per visit on average) and some laboratory tests. By following the current national guidelines, which are based on the 2001 WHO model, and subsequent recommendations related to HIV screening and mosquito nets distribution, public health centers can be seen to implement cost-effective ANC interventions. In contrast, the private clinic included in our study routinely included activities (in particular, three ultrasound scans) for which the evidence base is weak.

There was significant variation in efficiency in the provision of ANC services among our sample of facilities. The cost of providing a package of four ANC visits to one woman varied between $33 and $55 in the public facilities. Wide variation in antenatal care cost across health facilities implementing the same policy was similarly observed in Tanzania; from $2.78 to $59.48 [19]. In that study, variation was associated with the number of staff, structural and process quality of care, as well as the perceived quality of care. In our study, the observed variation in efficiency suggests that some health facilities have room for efficiency improvement. The most visited public health facility in our sample, Muhoza Health Center, presented the lowest unit cost. This suggests economies of scale, that is, if more women are mobilized to attend the clinic, the unit cost may be reduced. However, it is worth noting that this relationship was not present as a general trend throughout all the six health facilities.

The household cost of antenatal care appears at first sight rather small: transport accounts for 1% of the total cost of antenatal care and the time cost for women/couples accounts for 8% and 2% of the total in public and private facilities respectively. However, this level of the cost is meaningful in a country where 60.4% of the population earns less than $1.9 a day [27]. Transport cost can be a burden for the 15% of women/couples, who pay almost $1 for transport at each ANC visit. The amount of time for attending ANC clinic, including walking time to and from the clinic, was on average between four and five hours. This can be physically demanding for women in later months of pregnancy and a financial strain on poor families who rely on daily labour, particularly at the first visit, which partners are also expected to attend. Similar findings from Tanzania and India indicate that even when user fees are minimal or waived for maternal health services, the cost of transport and waiting time are still substantial [18, 28]. Long distance or transport difficulties to health facilities have been reported as a barrier to antenatal care utilization in Rwanda [29] and in other sub-Saharan countries [30–33], as has long waiting time [31, 34]. There is a need for a profound equity analysis of antenatal care utilization. The analysis should look at whether poor, vulnerable families have the same access to antenatal services as better-off families.

The micro-costing approach [35] used in this study is recommended for determining the cost of service interventions and for costing interventions using non-market goods, for which standard costs are not available [15]. Although our method was not exhaustive, we are confident that most resources used at each of the four visits prescribed in the national ANC guidelines have been correctly identified, measured and valued. Another strength of this study in comparison to many other ANC costing studies is the consideration of a societal perspective encompassing both health facility and household resource use.

The main methodological challenges in this study relate to data availability and the extent to which the chosen facilities represent the rest of the country. First, the cost of infrastructures was estimated using the standard cost of new government health center infrastructures to which a percentage of space from direct measurements of the space used to provide ANC in health facilities was applied. This was due to lack of information about the value and age of infrastructures. Second, the total number of ANC visits in surveyed health facilities in the year 2015 was not available in health facility reports or in national information systems. Therefore, this total number had to be estimated. For this purpose we used national figures for the first ANC registration from the national information system [17] (year 2015) for the surveyed facilities and the data on number of completed ANC visits obtained in our own cross-sectional household survey. Furthermore, the cost of ANC services was limited to the four standard ANC visits specified in the national and WHO guidelines. However, our data suggests that 4.5% of women/couples attended ANC more than four times. The cost of these additional visits was not included here. Third, unlike public health facilities, private health facilities vary in terms of the antenatal care package that is offered, as well as the size and quality of infrastructures and human resources. Thus one selected private health facility is unlikely to be representative of all private health facilities. To a lesser extent, the same concern regarding representation applies to public and faith-based health facilities, as our sample of five facilities is a small proportion of the nearly 500 health facilities in the country [36].

## Conclusion

Understanding the cost of current antenatal care practice is a necessary starting point for countries intending to revise their current ANC guidelines. The new WHO antenatal care guidelines recommend increasing the number of antenatal care contacts from the current four to eight. Our findings suggest that policy makers need to consider ways in which the non-financial barriers (especially the time cost) to attending ANC clinics can be reduced. This may include better organizing booking for appointments among other possible mechanisms. The distance to the clinic should also be reduced by looking at ways of bringing ANC services closer to the population, starting from areas where public transport is non-existent. The new WHO guidelines refer to “contacts” rather than “visits”, which may open up for other possible ways women can get in touch with the service provider. Yet such concerns must be balanced with women’s perception of the benefits of attending ANC, which may be reduced if personal contact is lacking. For Rwanda, the decision whether to adopt the new WHO guidelines or any other ANC reforms should take cost implications into consideration and the expected outcome gains from that reform, as well as the financial affordability (for both government and families) of those changes.

## Additional files

Additional file 1: Health facility interview guide. Additional file 1 is a tool that was used to collect cost data from six selected health facilities. It has six sections, from A to F. (DOCX 88 kb)

Additional file 2: Respondent characteristics and the number of ANC visits. Additional file 2 presents the result of analysis of Maternal Health Research in Rwanda (MatHeR), linked to this study, showing the respondent’s characteristics and number of antenatal care visits attended. (DOCX 22 kb)

Additional file 3: Cost-related questions from the household questionnaire. Additional file 3 presents an extract from a household questionnaire, used in data collection for the Maternal Health Research in Rwanda (MatHeR), which is linked to this study. This section is composed of questions related to antenatal care attendance, time use and payment for antenatal care. (DOCX 23 kb)

## Abbreviations

ANC: Antenatal care
HIV: Human Immunodeficiency Virus
WHO: World Health Organization
