# Supplementary material for: Cost of antenatal care for the health sector and for households in Rwanda
Source: BMC Health Serv Res. 2018 Apr 10;18:262. doi: 10.1186/s12913-018-3013-1 (PMC5891906; doi:10.1186/s12913-018-3013-1)
Supplement: Supplementary file 1 — Health facility interview guide. Additional file 1 is a tool that was used to collect cost data from six selected health facilities. It has six sections, from A to F. (DOCX 88 kb) [file 12913_2018_3013_MOESM1_ESM.docx]

**Additional file 1: Health facility interview guide**

**A. Identification of the health center**

Name of the health center

Location:

1. Province: …………………………….
2. District: …………………………….
3. Sector: …………………………….

**B. ANC general questions**

Does this Health center provide ANC service?

How many staffs are involved in the ANC service?

What is the academic qualification of the staff working in the ANC service?

How many days per week is the ANC service provided?

Does this Health center sometimes provide ANC service outside its premises? If Yes

| **Site1** | **Distance in Km from the HC** | **Days/month** | **Average number of women?** | **Means of transportation to the facility?** |
| --- | --- | --- | --- | --- |
|  |  |  |  |  |
|  |  |  |  |  |
|  |  |  |  |  |
|  |  |  |  |  |

Do you usually have activities for all women who came for ANC together (like education sessions)

What is the average time do you spend on such activities per day?

**C. ANC Specific Questions**

C.1. ANC first visit

| During the 1st ANC visit, do you perform the following activities ? | | | | | | | | | | | |
| --- | --- | --- | --- | --- | --- | --- | --- | --- | --- | --- | --- |
|  | Performed  Yes /No | Use Equipment | | Name of equipment | Use drug or consumable | | | Name of drugs and consumables | | | Average time |
|  |  | Yes | No |  | Yes | No | | Name | | Qty |  |
| Physical examination : check the general health state, measure blood pressure, weight, height… |  |  |  |  |  |  | |  | |  |  |
| Test breast |  |  |  |  |  |  | |  | |  |  |
| Gynecological examination : test abdomen… |  |  |  |  |  |  | |  | |  |  |
| HIV counseling |  |  |  |  |  |  | |  | |  |  |
| Delivery plan |  |  |  |  |  |  | |  | |  |  |
| Immunization against tetanus |  |  |  |  |  |  | |  | |  |  |
| Iron supplementation or provision of folic acid |  |  |  |  |  |  | |  | |  |  |
| Provide Mebendazole |  |  |  |  |  |  | |  | |  |  |
| Health education prevention of disease, nutrition… |  |  |  |  |  |  | |  | |  |  |
| Other |  |  |  |  |  |  | |  | |  |  |
| During the 1st ANC visit do you perform the following test? | | | | | | | | | | | |
|  | Performed ?  Yes or No | Equipment | | Consumables |  | | Average time | | Provider | | |
|  |  |  |  | Name | Qty | |  | |  | | |
| Haemoglobin test |  |  | |  |  | |  | |  | | |
| Syphilis test |  |  | |  |  | |  | |  | | |
| HIV test |  |  | |  |  | |  | |  | | |
| Blood type |  |  | |  |  | |  | |  | | |
| Urin test (albumin) |  |  | |  |  | |  | |  | | |
| Pregnancy test |  |  | |  |  | |  | |  | | |
| Glycemia |  |  | |  |  | |  | |  | | |
| Other test : explain |  |  | |  |  | |  | |  | | |

C.2. ANC Second visit

| During the 2^nd^ ANC visit, do you perform the following activities? | | | | | | | | | |
| --- | --- | --- | --- | --- | --- | --- | --- | --- | --- |
|  | Performed  Yes or No | Use Equipment | | Name of equipment | Use drug or consumable | | Name of drugs and consumables | | Average time |
|  |  | Yes | No |  | Yes | No | Name | Qty |  |
| Physical examination : check the general health state, measure blood pressure, weight, height… |  |  |  |  |  |  |  |  |  |
| Detect Malaria signs and other complications or disease |  |  |  |  |  |  |  |  |  |
| test breast |  |  |  |  |  |  |  |  |  |
| Gynecological examination: test abdomen (including BCF)… |  |  |  |  |  |  |  |  |  |
| Delivery plan |  |  |  |  |  |  |  |  |  |
| Immunization against tetanus |  |  |  |  |  |  |  |  |  |
| Provide iron supplementation or acide folique |  |  |  |  |  |  |  |  |  |
| Provide Mebendazole |  |  |  |  |  |  |  |  |  |
| Health education prevention of disease, nutrition, STIs, FP, new-born care, vaccination |  |  |  |  |  |  |  |  |  |

C. 3. ANC Third visit

| During the 3rd ANC visit, do you perform the following activities? | | | | | | | | | |
| --- | --- | --- | --- | --- | --- | --- | --- | --- | --- |
|  | Perfomed  Yes or No | Use equipment | | Name of equipment | Use drug or consumable | | Name of drugs and consumables | | Average time |
|  |  | Yes | No |  | Yes | No | Name | Qty |  |
| Physical examination: check the general health state, measure blood pressure, weight, height… |  |  |  |  |  |  |  |  |  |
| Detect Malaria signs and other complications or disease |  |  |  |  |  |  |  |  |  |
| test breast |  |  |  |  |  |  |  |  |  |
| Gynaecological examination : test abdomen (including BCF)… |  |  |  |  |  |  |  |  |  |
| Delivery plan |  |  |  |  |  |  |  |  |  |
| Immunization against tetanus |  |  |  |  |  |  |  |  |  |
| Provide iron supplementation or acide folique |  |  |  |  |  |  |  |  |  |
| Provide Mebendazole |  |  |  |  |  |  |  |  |  |
| Health education prevention of disease, nutrition, STIs, FP, new-born care, vaccination |  |  |  |  |  |  |  |  |  |
|  |  |  |  |  |  |  |  |  |  |

C.4. ANC fourth visit

| **During the 4th ANC visit, do you perform the following activities?** | | | | | | | | | |
| --- | --- | --- | --- | --- | --- | --- | --- | --- | --- |
|  | Perfomed  Yes or No | Use equipment | | | Use drug or consumable | | Name of drugs and consumables | | Average time |
|  |  | Yes | No | Name | Yes | No | Name | Qty |  |
| Physical examination: check the general health state, measure blood pressure, weight, height… |  |  |  |  |  |  |  |  |  |
| Detect Malaria signs and other complications or disease |  |  |  |  |  |  |  |  |  |
| test breast |  |  |  |  |  |  |  |  |  |
| Gynaecological examination: test abdomen (including BCF)… |  |  |  |  |  |  |  |  |  |
| Delivery plan |  |  |  |  |  |  |  |  |  |
| Immunization against tetanus |  |  |  |  |  |  |  |  |  |
| Provide iron supplementation or folic acid |  |  |  |  |  |  |  |  |  |
| Provide Mebendazole |  |  |  |  |  |  |  |  |  |
| Health education prevention of disease, nutrition, STIs, FP, new-born care, vaccination |  |  |  |  |  |  |  |  |  |
|  |  |  |  |  |  |  |  |  |  |

**SECTION D. Infrastructure cost**

|  | **Item** | **Quantity*** | **Cost** | **Useful life (yrs)** |
| --- | --- | --- | --- | --- |
|  | **Building cost** |  |  |  |
|  | Total building surface area (all floor in m^2^) |  |  |  |
|  | % allocation of space to ANC department |  |  |  |
|  | Age of building |  |  |  |
|  | Construction/purchase cost or  Monthly rent of building |  |  |  |
|  | **Equipment** |  |  |  |
|  |  |  |  |  |
|  |  |  |  |  |
|  |  |  |  |  |
|  |  |  |  |  |
|  |  |  |  |  |
|  |  |  |  |  |
|  |  |  |  |  |
|  |  |  |  |  |
|  |  |  |  |  |
|  | **Furniture** |  |  |  |
|  |  |  |  |  |
|  |  |  |  |  |
|  |  |  |  |  |
|  |  |  |  |  |
|  |  |  |  |  |
|  | **Materials &supplies** |  |  |  |
|  |  |  |  |  |
|  |  |  |  |  |
|  |  |  |  |  |
|  |  |  |  |  |
|  |  |  |  |  |
|  |  |  |  |  |
|  |  |  |  |  |
|  |  |  |  |  |
|  |  |  |  |  |
|  |  |  |  |  |

SECTION E. Record on utilities

| **No.** | **Items** | **Quantity** | **Unit of measurement** | **Total expenditure** | **No. of months covered** |
| --- | --- | --- | --- | --- | --- |
| 1. | **Means of transport (Outreach, if applicable)** |  |  |  |  |
|  | Lubricant |  |  |  |  |
|  | Maintenance |  |  |  |  |
|  | Repairs |  |  |  |  |
|  | Insurance |  |  |  |  |
|  | Tire spare parts |  |  |  |  |
|  | Others |  |  |  |  |
| 2. | **Building** |  |  |  |  |
|  | Electricity |  |  |  |  |
|  | Water |  |  |  |  |
|  | Maintenance |  |  |  |  |
|  | Telephone |  |  |  |  |
|  | Cleaning |  |  |  |  |
|  | Other |  |  |  |  |
| 3 | **Equipment** |  |  |  |  |
|  | Maintenance |  |  |  |  |
|  | Repairs |  |  |  |  |
|  | Others |  |  |  |  |

SECTION F. Personnel in ANC unit

| **Staff no.** | **Job title^1^** | **1=Full time**  **2= Part time** | **What percent of his/her time goes into ANC? (%)** | **Main task^2^** | **In last one week, how much time was spent in handling:** | | | | **Salary and other benefits** |
| --- | --- | --- | --- | --- | --- | --- | --- | --- | --- |
|  |  |  |  |  | **ANC 1^st^ visit** | **ANC 2^nd^ visit** | **ANC 3^rd^ visit** | **ANC 4^th^ visit** |  |
|  |  |  |  |  |  |  |  |  |  |
|  |  |  |  |  |  |  |  |  |  |
|  |  |  |  |  |  |  |  |  |  |
|  |  |  |  |  |  |  |  |  |  |
|  |  |  |  |  |  |  |  |  |  |
|  |  |  |  |  |  |  |  |  |  |
|  |  |  |  |  |  |  |  |  |  |
|  |  |  |  |  |  |  |  |  |  |
|  |  |  |  |  |  |  |  |  |  |
|  |  |  |  |  |  |  |  |  |  |

| ^1^ Fill in the relevant (code) from the list below for job title   1. Nurse A0 2. Nurse A1 3. Nurse A2 4. Midwife 5. Medical Officer 6. Gynecologist 7. Lab technician 8. Trained Administrative assistant 9. Untrained Office assistant 10. Sweeper 11. Social worker 12. Other ……….. | ^2^ Fill in the relevant (code ) from the list below  1. Reception and registration  2. Physical examination  3. Counseling  4. Laboratory  5. Health Education  6. Dispensing drugs  7. Cleaning  8. Driving  9. Administrative procedures |
| --- | --- |
