# Supplementary material for: Cost of antenatal care for the health sector and for households in Rwanda
Source: BMC Health Serv Res. 2018 Apr 10;18:262. doi: 10.1186/s12913-018-3013-1 (PMC5891906; doi:10.1186/s12913-018-3013-1)
Supplement: Supplementary file 2 — Respondent characteristics and the number of ANC visits. Additional file 2 presents the result of analysis of Maternal Health Research in Rwanda (MatHeR), linked to this study, showing the respondent’s characteristics and number of antenatal care visits attended. (DOCX 22 kb) [file 12913_2018_3013_MOESM2_ESM.docx]

**Additional file 2: Respondent characteristics and the number of ANC visits (MatHeR study)**

|  | **Number of ANC visits** | | | | | |
| --- | --- | --- | --- | --- | --- | --- |
|  | **1 visit** | **2 visits** | **3 visits** | **4 visits** | **5+ visits** | **Total** |
| **Province** |  |  |  |  |  |  |
| Northern Province | 16 | 68 | 288 | 303 | 26 | 701 |
| Kigali city | 7 | 26 | 92 | 77 | 11 | 213 |
| **Total** | **23** | **94** | **380** | **380** | **37** | **914** |
|  |  |  |  |  |  |  |
| **Main daily activity** |  |  |  |  |  |  |
| Paid job | 0 | 3 | 12 | 14 | 6 | 35 |
| Student | 0 | 3 | 12 | 4 | 1 | 20 |
| Housewife | 2 | 7 | 30 | 27 | 2 | 68 |
| Work in the field | 13 | 57 | 250 | 291 | 23 | 634 |
| Own business | 1 | 7 | 27 | 12 | 1 | 48 |
| No activity | 4 | 12 | 42 | 25 | 3 | 86 |
| Others | 3 | 5 | 7 | 6 | 1 | 22 |
| **Total** | **23** | **94** | **380** | **379** | **37** | **913** |
|  |  |  |  |  |  |  |
| **Age** |  |  |  |  |  |  |
| 15-20 | 0 | 13 | 28 | 46 | 5 | 92 |
| 21-25 | 4 | 22 | 120 | 105 | 14 | 265 |
| 26-30 | 6 | 22 | 102 | 131 | 9 | 270 |
| 31-35 | 9 | 18 | 80 | 58 | 8 | 173 |
| 36-40 | 2 | 16 | 35 | 30 | 1 | 84 |
| >40 | 2 | 3 | 14 | 10 | 0 | 29 |
| **Total** | **23** | **94** | **379** | **380** | **37** | **913** |
|  |  |  |  |  |  |  |
| **Number of children** |  |  |  |  |  |  |
| One child | 0 | 4 | 15 | 19 | 3 | 41 |
| Two children | 7 | 20 | 98 | 95 | 5 | 225 |
| Three children | 4 | 14 | 64 | 63 | 3 | 148 |
| 4-5 children | 4 | 18 | 55 | 58 | 3 | 138 |
| 6 children or more | 5 | 12 | 40 | 19 | 1 | 77 |
| **Total** | **20** | **68** | **272** | **254** | **15** | **629** |
|  |  |  |  |  |  |  |
| **Educational level** |  |  |  |  |  |  |
| Primary but not complete | 10 | 47 | 182 | 165 | 10 | 414 |
| Primary level | 4 | 15 | 76 | 101 | 6 | 202 |
| Lower secondary or vocational | 3 | 11 | 45 | 45 | 6 | 110 |
| Upper secondary or higher education | 1 | 10 | 41 | 35 | 11 | 98 |
| **Total** | **18** | **83** | **344** | **346** | **33** | **824** |
|  |  |  |  |  |  |  |
| **Marital status** |  |  |  |  |  |  |
| Married | 9 | 42 | 196 | 214 | 20 | 481 |
| Cohabitant | 8 | 24 | 122 | 121 | 15 | 290 |
| Separated/Divorced/Widowed | 3 | 3 | 11 | 6 | 0 | 23 |
| Not married/Single | 3 | 25 | 51 | 38 | 2 | 119 |
| **Total** | **23** | **94** | **380** | **379** | **37** | **913** |
