# Supplementary material for: Cost of antenatal care for the health sector and for households in Rwanda
Source: BMC Health Serv Res. 2018 Apr 10;18:262. doi: 10.1186/s12913-018-3013-1 (PMC5891906; doi:10.1186/s12913-018-3013-1)
Supplement: Supplementary file 3 — Cost-related questions from the household questionnaire. Additional file 3 presents an extract from a household questionnaire, used in data collection for the Maternal Health Research in Rwanda (MatHeR), which is linked to this study. This section is composed of questions related to antenatal care attendance, time use and payment for antenatal care. (DOCX 23 kb) [file 12913_2018_3013_MOESM3_ESM.docx]

**Additional file 3: Cost-related questions from the household questionnaire (MatHeR study)**

**ANTENATAL CARE**

**C 14. Did you go to the antenatal care clinic (ANC) for check-ups during your latest pregnancy (index child)?**

1. Yes
2. No

**C 15. If YES, how many visits did you do at the ANC clinic during your latest pregnancy (index child)?**

1. 1 visit
2. 2 visits
3. 3 visits
4. 4 visits
5. More than 4 visits

**C 16. Did you go anywhere else for check-ups of your pregnancy (index child)?**

1. Yes
2. No

**C 17. If YES, where did you go?**

1. To a traditional birth attendant (TBA) or a Traditional Healer

2. To the community health worker (CHW)

3. To any other village health provider

4. To a friend or family member

**If the respondent never attended the antenatal care during the index pregnancy, please jump to question D1.**

**If the respondent visited the ANC clinic at least once, please continue with the questions below.**

**C 18. The ANC clinic that you visited, was it located in …**

1. A health post/dispensary
2. A health centre
3. A district hospital /provincial hospital
4. A referral hospital
5. Any other health facility
6. A private clinic

**C19. Did you commonly pay for ANC service?**

1. No
2. Yes – how much?....................RWF

**C 20. During your latest pregnancy (index child), when did you go to see the staff at the ANC clinic and whom did you meet, a nurse/midwife (=1), a doctor (=2) or any other health professional (=3); and who went with you to the ANC clinic?**

*(Please fill in data for each visit, when it took place, who the care-giver was and who accompanied the woman to the clinic).*

| **ANC visits** | **1-3 months of pregnancy** | **4-6 months of pregnancy** | **7 months to end of pregnancy** | **Who was the health provider?**  *Mark with:*  *1= nurse/midwife*  *2= doctor*  *3= other health professional* | **Who went with you to the ANC clinic?**  *Mark with:*  *1=Husband*  *2=Mother*  *3=Family member*  *4=CHW*  *5=Other person*  *6= No one* |
| --- | --- | --- | --- | --- | --- |
| First visit |  |  |  |  |  |
| Second visit |  |  |  |  |  |
| Third visit |  |  |  |  |  |
| Fourth visit |  |  |  |  |  |
| Fifth visit |  |  |  |  |  |
| Sixth visit |  |  |  |  |  |
| Seventh |  |  |  |  |  |

**C 21. What mode of transport did you commonly use to get to the ANC clinic?**

1. Walking
2. Bicycle
3. Motorbike
4. Bus
5. Car

**C 22. Did you commonly have to pay for the transport to the ANC clinic?**

1. No
2. Yes, I paid……………….. RWF

**C 23. How long time did it commonly take you to get to the ANC clinic?**

1. 5- 30 minutes
2. 30-60 minutes
3. 1-2 hours
4. 2-3 hours
5. More than 3 hours

**C 24. How long time did you commonly spend on travelling to the ANC clinic, attend the service and travel back?................................ hours**
